# Maternal Metabolome in Pregnancy and Childhood Asthma or Recurrent Wheeze in the Vitamin D Antenatal Asthma Reduction Trial

**DOI:** 10.3390/metabo11020065

**Published:** 2021-01-23

**Authors:** Mengna Huang, Rachel S. Kelly, Su H. Chu, Priyadarshini Kachroo, Gözde Gürdeniz, Bo L. Chawes, Hans Bisgaard, Scott T. Weiss, Jessica Lasky-Su

**Affiliations:** 1 Channing Division of Network Medicine, Department of Medicine, Brigham and Women’s Hospital and Harvard Medical School, Boston, MA 02115, USA; mengna.huang@channing.harvard.edu (M.H.); rachel.kelly@channing.harvard.edu (R.S.K.); su.chu@channing.harvard.edu (S.H.C.); priyadarshini.kachroo@channing.harvard.edu (P.K.); scott.weiss@channing.harvard.edu (S.T.W.); 2 Copenhagen Prospective Studies on Asthma in Childhood (COPSAC), Herlev and Gentofte Hospital, University of Copenhagen, 2820 Copenhagen, Denmark; gozde.gurdeniz@dbac.dk (G.G.); chawes@copsac.com (B.L.C.); bisgaard@copsac.com (H.B.)

**Keywords:** metabolomic epidemiology, pregnancy metabolome, childhood asthma, maternal child health, Vitamin D Antenatal Asthma Reduction Trial (VDAART)

## Abstract

The in utero environment during pregnancy has important implications for the developing health of the child. We aim to examine the potential impact of maternal metabolome at two different timepoints in pregnancy on offspring respiratory health in early life. In 685 mother-child pairs from the Vitamin D Antenatal Asthma Reduction Trial, we assessed the prospective associations between maternal metabolites at both baseline (10–18 weeks gestation) and third trimester (32–38 weeks gestation) and the risk of child asthma or recurrent wheeze by age three using logistic regression models accounting for confounding factors. Subgroup analyses were performed by child sex. Among 632 metabolites, 19 (3.0%) and 62 (9.8%) from baseline and third trimester, respectively, were associated with the outcome (*p*-value < 0.05). Coffee-related metabolites in the maternal metabolome appeared to be of particular importance. Caffeine, theophylline, trigonelline, quinate, and 3-hydroxypyridine sulfate were inversely associated with asthma risk at a minimum of one timepoint. Additional observations also highlight the roles of steroid and sphingolipid metabolites. Overall, there was a stronger relationship between the metabolome in later pregnancy and offspring asthma risk. Our results suggest that alterations in prenatal metabolites may act as drivers of the development of offspring asthma.

## 1. Introduction

The in utero environment has a critical impact on the developing health of the child throughout early life. However, the mechanistic pathobiology underlying this relationship is not yet fully understood. The in utero environment experienced by a fetus results from a complex interplay between maternal health, exposure, and genetics. As such, the metabolome, which reflects the downstream “net results” of genetic, transcriptomic, proteomic, and environmental interactions [1], is ideally suited to explore the prenatal influence of a mother on the health of her child.

Evidence suggests the development of a child’s respiratory system is particularly susceptible to perturbations during the prenatal period, and there is likely a critical window during development of the respiratory system that is most important for the risk of future respiratory disease [2]. Both human and experimental data have shown that a perturbed prenatal environment, particularly as a result of maternal exposure to xenobiotics, can alter immune system development and postnatal function [3]. Prenatal reprogramming, therefore, plays a key role in the incidence of respiratory conditions such as asthma among offspring [4].

Asthma represents a significant public health burden, with the majority of cases originating in early life. Globally, it is the most common chronic disease in children with increasing prevalence, and as such, is the cause of considerable morbidity and mortality, particularly when asthma persists into adulthood [5,6]. Therefore, improving the understanding of, and identifying modifiable components within, the critical windows of prenatal development could support the discovery of novel strategies to ameliorate risk of childhood respiratory conditions and reduce the burden of asthma worldwide.

Given the role of the metabolome as a reflection of a mother’s genetics, environment and their interactions on her child’s risk of asthma, and the fact that elements of the metabolome may be modifiable through diet, supplementation, and other interventions, metabolomics represents the ideal technology to explore the in utero environment’s influence on childhood asthma. In this study, we aim to explore how the maternal plasma metabolome may impact the risk of asthma in offspring, leveraging mother-child pairs from the Vitamin D Antenatal Asthma Reduction Trial (VDAART).

## 2. Results

A total of 685 mother-child pairs from VDAART were eligible for inclusion in this study (Supplementary Figure S1). Of these, 200 (29.2%) children were classified as having asthma or recurrent wheeze by age three (for definition see Section 4.2.). The mothers of children who developed asthma/recurrent wheeze were younger than those whose children did not (Table 1). They had lower vitamin D levels at baseline and in the third trimester, and were more likely to have asthma themselves, and more likely to be black and in a lower income and educational category. The mothers of children who did not develop asthma/recurrent wheeze were more likely to live in San Diego.

In total, 632 metabolites were measured and passed quality control in the plasma samples from both baseline and third trimester. A majority of these metabolites were lipids (52.8%) and amino acids (23.4%). In the maternal baseline (10–18 weeks gestation) plasma samples, 19 (3.0%) metabolites were associated with asthma/recurrent wheeze by age three in offspring (*p*-value < 0.05) after accounting for maternal characteristics including age at baseline, race (white, black, others), asthma status, educational level, household income, exact baseline gestational week, blood vitamin D level at the time of blood draw, trial treatment, and study site. These metabolites were primarily lipids (*n* = 10) and xenobiotics (*n* = 5), and the majority (*n* = 14, 73.7%) were positively associated with risk (Supplementary Table S1). Only one metabolite, 2-aminoadipate, an intermediate of lysine metabolism, was significant at an ENT90% (effective number of independent tests accounting for 90% of the total variance in metabolites, see Section 4.4.) threshold (odds ratio (OR): 1.84, 95% confidence interval (CI): 1.37, 2.47, *p*-value = 5.24 × 10^−5^). In the third trimester samples, a much larger number of metabolites, 62 (9.8%), were significant (*p*-value < 0.05) after adjusting for the same set of confounders, and again most of these (*n* = 41, 66.1%) were at higher levels in those women whose children went on to develop asthma/recurrent wheeze (Supplementary Table S2). Of the 62 metabolites 35 were lipids, including nine metabolites involved in sphingolipid metabolism. The top hit, *N*-palmitoylglycine, an acylglycine, was the only metabolite to reach significance at an ENT90% threshold (OR: 2.01, 95% CI: 1.39, 2.91, *p*-value = 2.10 × 10^−4^).

**Figure 1:**
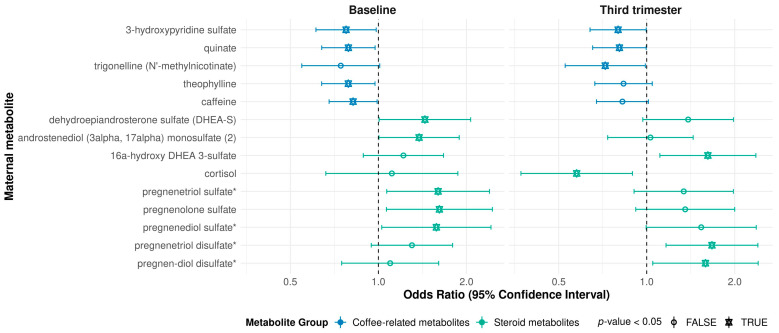
Coffee-related and steroid metabolites in the maternal metabolome significantly associated with risk of age three asthma/recurrent wheeze at either baseline or third trimester. * in metabolite names indicates compounds that have not been officially confirmed based on a standard, but we are confident in its identity.

Only four metabolites were significantly associated with offspring asthma risk at both baseline and in the third trimester. Increased levels of the tyrosine metabolite thyroxine (OR: 2.10, 95% CI: 1.07, 4.12, *p*-value = 3.21 × 10^−2^ at baseline; OR: 1.91, 95% CI: 1.10, 3.33, *p*-value = 2.26 × 10^−2^ at third trimester), and sphingomyelin (d18:2/24:1, d18:1/24:2) (OR: 2.01, 95% CI: 1.06, 3.79, *p*-value = 3.25 × 10^−2^ at baseline; OR: 2.12, 95% CI: 1.10, 4.09, *p*-value = 2.57 × 10^−2^ at third trimester) in the mothers at both baseline and the third trimester were associated with an increased risk of asthma in their offspring. Two xenobiotics, quinate and 3-hydroxypyridine sulfate, both of which have been associated with coffee intake [7,8,9,10], were inversely associated with asthma risk at both timepoints. Coffee related metabolites in the maternal metabolome appeared to be of particular importance in the risk of offspring asthma. Two other coffee metabolites [7,8,9,10,11] that were inversely associated with risk in the baseline samples, caffeine and theophylline were approaching significance in the third trimester. Trigonelline (*N*′-methylnicotinate), which has been suggested as a biomarker of coffee consumption [7,8,9,10], was significant in the third trimester, and borderline significant at baseline (Figure 1, Supplementary Tables S1 and S2).

A similar pattern emerged for the steroid metabolites (Figure 1). Although different metabolites were significant at the two measured timepoints, overall higher maternal levels of steroid metabolites across the course of pregnancy were associated with increased risk of childhood asthma. Only cortisol appeared to have an inverse association in the third trimester (OR: 0.58, 95% CI: 0.37, 0.89, *p*-value = 1.38 × 10^−2^).

**Figure 2:**
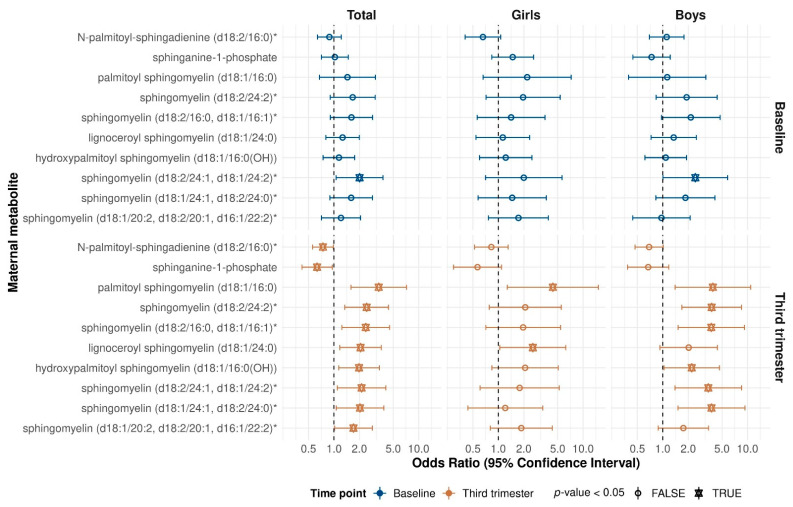
Results for sphingolipid metabolites from both the maternal timepoints that were significantly associated with risk of age three asthma/recurrent wheeze in analysis of the third trimester metabolome; overall and stratified by child sex. * in metabolite names indicates compounds that have not been officially confirmed based on a standard, but we are confident in its identity.

Given known sex-differences in childhood asthma, we further stratified our results by child sex. Among 358 boys, 121 (33.8%) went on to develop asthma/recurrent wheeze by age 3, whereas 79 out of the 327 girls (24.2%) did so. As in the total population, the strongest association with offspring asthma was with the third trimester metabolome, which was most evident for sphingolipid biosynthesis (Figure 2), and there were few common metabolites between the two timepoints in either sex. At both timepoints, there were more significant associations among the girls than the boys; 32 (Supplementary Table S3) versus 23 (Supplementary Table S4) for baseline, and 52 (Supplementary Table S5) versus 47 (Supplementary Table S6) for third trimester. The directions of effect tended to be consistent between the sexes even if a given metabolite did not reach significance in one or the other.

To further explore the potential role of offspring sex as a potential effect modifier, we searched for interactions with maternal metabolite levels in the risk of childhood asthma. A large number of metabolites demonstrated a significant interaction at both timepoints (Supplementary Tables S7 and S8). While the exact metabolites differed, both showed a role for tryptophan and xanthine metabolism. Among the tryptophan metabolites, kynurenine and kynurenate appeared to statistically interact with offspring sex. Among the xanthine metabolites, theobromine demonstrated a significant interaction with sex at both baseline (interaction *p*-value = 7.72 × 10^−3^) and third trimester (interaction *p*-value = 3.76 × 10^−2^). It was inversely associated with risk in the female offspring, but positively (and non-significantly) associated with risk in male offspring. Like caffeine and theophylline, theobromine is a xanthine alkaloid, and one of the caffeine metabolites. Another metabolite of xanthine metabolism, 3-methylxanthine, also demonstrated a significant interaction at both timepoints. While caffeine, theophylline and 1,7-dimethylurate demonstrated significant interactions in the third trimester only.

## 3. Discussion

While there are a growing number of studies investigating the role of the metabolome in asthma [12], to date, none have considered the impact of the global maternal metabolome in pregnancy on the risk of asthma in her offspring. The developmental origins hypothesis states that the in utero environment has an impact on fetal development and childhood health [13]. A mother’s health, exposures, and genetics influence that environment and are reflected in her metabolomic profile, which is representative of her physiological state throughout pregnancy [13]. There is an increasing body of literature demonstrating that the metabolome during pregnancy is associated with newborn outcomes, such as birth weight, small-for-gestational-age status, and hyperinsulinaemia [13,14,15]. Given that we know the in utero environment is crucial to the development of respiratory health, and that asthma is a whole system disorder that is reflected in the metabolome, in this study, we aimed to determine if and how the maternal metabolome associates with the risk of early life asthma or recurrent wheeze.

In this study, we leveraged 685 mother-child pairs from VDAART, a vitamin D prenatal supplementation trial for the prevention of asthma [16]. We performed metabolomic profiling on plasma samples from the mothers collected at two timepoints across pregnancy, at study recruitment/baseline (10–18 weeks gestation) and in the third trimester (32–38 weeks gestation). We then searched for metabolites at these two timepoints which associated with risk of asthma/recurrent wheeze by age three in offspring. Our results indicated that components of the plasma metabolome, which can be considered a proxy for the in utero environment, are associated with offspring asthma risk. These component metabolites were largely different at baseline as compared to third trimester, although in many cases, they were involved in the same biological pathways and processes. This is not perhaps to be expected as the in utero environment throughout pregnancy is known to have differing effects on fetal development [17]. Perhaps unsurprisingly, there was a stronger relationship between the maternal metabolome in later pregnancy and offspring asthma.

Our findings in this current study, which is based in blood, support a role for the maternal diet in the association between maternal metabolome and offspring asthma. A number of the significant hits from both baseline and third trimester have been identified to be metabolites of coffee or correlated with coffee intake, including caffeine, theophylline, quinate, trigonelline, and 3-hydroxypyridine sulfate [7,8,9,10]. Interestingly, the World Health Organization recommends limiting coffee intake during pregnancy, as it has been associated with pre-term birth, reduced birth weight, and pregnancy loss [18,19,20]. Nevertheless, the observed protective effects of coffee metabolites in this study are in line with evidence suggesting a possible inverse association between coffee consumption during pregnancy and asthma in the offspring [21]. These protective effects may relate to the anti-inflammatory and immunomodulatory properties of caffeine, or its ability to increase expression of surfactant protein (SP)-B, which crucial for the physiological function of pulmonary surfactant [21,22]. Theophylline also has demonstrated anti-inflammatory properties and was previously used clinically as a bronchodilator, although its usage has now largely been discontinued due to associated side effects [23]. Unfortunately, we do not have information on coffee consumption for the mothers in this cohort, and it is possible these metabolites came from other sources, such as chocolate or tea, but these findings suggest further work may be warranted to explore the effects of coffee and its metabolites during pregnancy on the health of the offspring. Common to the theme of inflammation, the top hit in baseline maternal metabolome, 2-aminoadipate, has been previously implicated in type 2 diabetes [24], as a lysine oxidation product by myeloperoxidase in inflammatory processes [25], whereas *N*-palmitoylglycine (top hit in third trimester maternal metabolome) may stimulate the production of nitric oxide [26], which is involved in asthma physiopathology [27].

Further evidence that the maternal metabolome may influence childhood asthma comes from the known link between maternal diet and childhood asthma [28]. In particular, it has been hypothesized maternal diet, and the bacterial metabolites influenced by diet, may affect asthma via altered transcription of certain FOXP3 genes in the lung, which are associated with the development and function of T regulatory cells and are known to affect the development of asthma [28,29]. A high intake of fiber may be especially protective due to the resulting excess generation of acetate, the predominant metabolite produced from fiber by gut bacteria and the most abundant short chain fatty acid in the body [28]. It is thought this may be due to its ability to regulate the function of T regulatory cells [30]. Within the VDAART cohort, we have previously reported that mothers with higher intestinal levels of acetic acid, from which acetate is formed, in their third trimester were less likely to have offspring with asthma/recurrent wheeze, and that the abundance of acetic acid arose from the interactions between both dietary fiber and the composition of their microbiome [31].

We also identified an important role for steroids. With the exception of cortisol, higher levels of maternal steroids, particularly pregnenolone steroids, were associated with an increased risk of offspring asthma. Given that VDAART mothers were recruited based on the condition of having asthma, eczema, or allergic rhinitis, this is of particular interest. Corticosteroids administered either orally or via inhalation are a first-line treatment for asthma, due to their anti-inflammatory properties which act to reduce mucosal edema and bronchial hyperreactivity thus relieving acute symptoms and preventing structural damage to the lungs [32]. The positive association between steroids and offspring in asthma is, therefore, somewhat counter-intuitive, although we did see an inverse relationship with cortisol which is in agreement with other studies [32]. The relationship between steroids in the maternal metabolome and offspring asthma may be particularly complex because pregnancy is a transient period of hypercortisolism, as the placenta-derived corticotropin-releasing hormone (CRH) progressively increases in the maternal circulation, resulting in increased circulating cortisol levels. This is especially evident during the third trimester, which may explain our differing results for cortisol at this time period as compared to baseline.

We further observed that ten third trimester sphingolipids were significantly associated with risk of offspring asthma or recurrent wheeze, among which eight were sphingomyelins. An increasing body of work supports a role for sphingolipid metabolism in asthma and poor respiratory health [33,34]. Sphingolipids are key structural elements in cellular membranes and essential signaling molecules for multiple cellular functions including immune responses, due to their ability to form multiple hydrogen bonds with other molecules [35,36]. Sphingolipids and the key sphingolipid metabolism mediator, sphingosine-1-phosphate have been implicated in asthma due to their actions on airway smooth muscle cell hyper-responsiveness, lung inflammation, and mast cell activation [34]. Sphingomyelins represent the dominant sphingolipids in the mammalian membrane and are particularly involved in the regulation of endocytosis and receptor-mediated ligand uptake, and in ion channel and G-protein-coupled receptor function [35]. The sphingomyelins were positively associated with risk, which is in agreement with work demonstrating that children with allergic asthma had higher levels of sphingomyelins than controls. Although intriguingly, this same study found that children with non-allergic asthma had lower sphingomyelins than controls [33]. *N*-palmitoyl-sphingadienine (d18:2/16:0), which is a long-chain dienic base of human plasma sphingomyelins, and sphinganine-1-phosphate, an intermediate in the metabolism of sphingolipids were inversely associated with risk in this study. It has been reported that de novo synthesis of sphingolipids is reduced in children with asthma [33]. These findings may be a reflection of the complexity of sphingolipid metabolism, where conversions between ceramides and different subclasses of sphingolipids may be possible [36].

There were a number of limitations to these analyses. The metabolomics profiling for the baseline and third trimester samples was conducted at two different timepoints. Given the relative nature of untargeted metabolomic profiling, they could not be combined. This meant we could not directly compare or track levels of the metabolites of interest across pregnancy. Information on fasting status at plasma collection was not collected. We were also not measuring the level of these metabolites in the amniotic fluid or in the fetus but indirectly in maternal blood. Finally, the majority of our metabolites did not pass stringent correction for multiple testing, and there is a potential for false positive findings. Nevertheless, this study, which was conducted within a large well-characterized mother-child cohort, including a multi-ethnic population, is the first of its kind to study the maternal metabolome and childhood asthma, and as such, should be considered hypothesis-generating. Further research in independent populations with metabolomic data from similar stages of pregnancy is warranted to further explore our significant hits, which have a biologically plausible relationship with asthma/recurrent wheeze development.

## 4. Materials and Methods

### 4.1. Study Subjects

The Vitamin D Antenatal Asthma Reduction Trial (VDAART) was a randomized, double-blind, parallel-design trial conducted at three study sites across the United States (ClinicalTrials.gov identifier: NCT00920621), to determine whether prenatal vitamin D supplementation lowers the risk of asthma in offspring. Details on study rationale, design, methods, and results have been published elsewhere [16]. Briefly, between October 2009 and July 2011, VDAART recruited pregnant non-smoking women aged 18–39 years who had a history of asthma, eczema, or allergic rhinitis, or whose partner (biologic father of the child) had a history of these conditions. At 10–18 weeks gestation, 440 women were randomized to 4000 IU vitamin D daily, while 436 women were randomized to a daily placebo (both arms also received daily prenatal multivitamin containing 400 IU vitamin D). The outcome of interest was the composite measure of asthma or recurrent wheeze by age three years as described previously [16]. The institutional review boards at each participating Clinical Center and the Data Coordinating Center at Brigham and Women’s Hospital approved protocols of the trial, with informed consent obtained from pregnant women at the enrollment visit covering both primary and secondary analyses of data. The current analysis included women with plasma metabolomic data at two timepoints: study baseline (10–18 weeks gestation) and third trimester (32–38 weeks gestation), and their children, resulting in a final sample size of 685 mother-child pairs.

### 4.2. Asthma or Recurrent Wheeze by the Age of Three Years in Children

The primary outcome of the present analysis, childhood asthma or recurrent wheeze by age three, was defined in the same way as the primary endpoint of the trial [16]. It is the composite of asthma, defined as a parental report of physician-diagnosed asthma, and recurrent wheeze, defined as a parental report of recurrent wheeze in the child’s first three years of life, where recurrent wheeze is the occurrence of at least one of the following five conditions: (1) parental report of wheeze after child’s second birthday with at least one report of wheeze prior to second birthday; (2) report of child’s use of asthma controller medication (steroid inhalers or nebulizers, leukotriene modifiers, or steroid pills or liquids) after the second birthday, with a report of wheeze before the second birthday; (3) two or more distinct parental reports of wheeze after the second birthday; (4) one or more parental report of wheeze and use of asthma controller medications at distinct visits, both after the second birthday; or (5) two or more distinct reports of use of asthma controller medications after the second birthday.

### 4.3. Metabolomics Data

Global metabolomic profiles of VDAART mothers were obtained using ultrahigh-performance liquid-chromatography (UPLC) coupled with tandem mass spectrometry (MS/MS) at Metabolon Inc. and an untargeted approach [37]. Blood samples were collected in EDTA tubes and centrifuged at 2000 RPM at 4 °C, after which plasma was separated and stored at −80 °C until processing. During data processing and quality control, we imputed missing metabolite values by replacement with half of the lowest observed value in all samples for each metabolite. Metabolite features were then log-10 transformed and *pareto*-scaled. Six hundred and thirty-two known metabolites common to both timepoints in pregnancy with less than or equal to 30% missing before imputation were included for analyses. The metabolomic assay, data processing, and quality control processes are detailed in the Supplementary File. Because the metabolomic profiling of the baseline and third trimester samples was conducted separately, the relatively quantified data cannot be combined.

### 4.4. Statistical Analysis

We summarized the characteristics of VDAART mothers with metabolomic data at both baseline and third trimester according to whether their children developed asthma or recurrent wheeze by the age of three years. Bivariate significance was tested using chi-squared test for categorical variables and two sample *t*-test for continuous variables. Logistic regression models were used to assess the associations between each maternal plasma metabolite (as the independent variable) and children’s asthma or recurrent wheeze status by age three (as the dependent variable), for both timepoints in pregnancy respectively, adjusting for potential confounding factors. Potential confounding factors were selected based on scientific literature considering their causal relations with the metabolites and with the risk of childhood asthma or wheeze. The primary models adjusted for maternal characteristics including age at baseline, race (white, black, others), asthma status (no asthma, controlled asthma, any uncontrolled asthma during prenatal visits), educational level (college graduate or higher, some college, high school or technical school, less than high school), household income (<$30,000, $30,000–$74,999, $75,000–$99,999, ≥$100,000, prefer not to answer/do not know), exact baseline gestational week, blood vitamin D level at the time of blood draw, trial treatment, and study site (Boston, St. Louis, San Diego). We ran additional models stratifying by child sex (female/male) to evaluate potential effect modification, adjusting for the same set of potential confounding factors. We also examined whether there were interactions with child sex by including interaction terms in the logistic regression models. To account for multiple comparisons while taking into consideration the high correlation between metabolites that exist within interconnected pathways, we applied the effective number of independent test (ENT) approach [38,39], exploring a threshold of ENT90% (accounting for 90% of the total variance in metabolites; corresponding *p*-value thresholds were 3.07 × 10^−4^ at baseline and 3.05 × 10^−4^ at third trimester). All analyses are conducted in R version 3.6.3 [40].

## 5. Conclusions

In this study, we identified a number of metabolites in the maternal metabolome, including those involved in coffee, steroid, and sphingolipid metabolism, which may be associated with the risk of asthma in offspring (Supplementary Figure S2). The majority of these metabolites, as well as others of interest in this study, such as tryptophan, have previously been associated with asthma [41]. This work suggests that metabolites associated with asthma status may also act as drivers of the development of asthma during the prenatal period. Our results may help with the development of novel prenatal preventative strategies to decrease the incidence of childhood asthma.

## Figures and Tables

**Table 1:** Characteristics of mothers with 10–18 weeks (baseline) & 32–38 weeks gestation (third trimester) metabolomics data by children’s status of asthma or recurrent wheeze by age three.

	No Asthma/Wheeze by Age 3 (*n* = 485)	Asthma/Wheeze by Age 3 (*n* = 200)	*p*-Value
Maternal Characteristics			
Age, mean (SD)	27.9 (5.3)	26.1 (5.6)	<0.001
Pre-pregnancy BMI, mean (SD)	28.2 (7.5) ^a^	29.0 (9.0) ^b^	0.323
Baseline vitamin D level, mean (SD)	23.4 (10.5)	21.2 (9.6)	0.008
Third trimester vitamin D level, mean (SD)	34.0 (14.4)	30.4 (15.1)	0.004
Exact baseline gestational week, mean (SD)	14.0 (2.7)	14.5 (2.8)	0.032
Treatment group, *n* (%)			0.140
4400 IU/day vitamin D	250 (51.5%)	90 (45.0%)	
400 IU/day vitamin D	235 (48.5%)	110 (55.0%)	
Site, *n* (%)			0.003
Boston	126 (26.0%)	67 (33.5%)	
San Diego	178 (36.7%)	47 (23.5%)	
St Louis	181 (37.3%)	86 (43.0%)	
Race, *n* (%)			0.002
Black	195 (40.2%)	109 (54.5%)	
Other	82 (16.9%)	23 (11.5%)	
White	208 (42.9%)	68 (34.0%)	
Asthma, *n* (%)			<0.001
Yes	177 (36.5%)	104 (52.0%)	
No	308 (63.5%)	96 (48.0%)	
Education level, *n* (%)			0.013
≥College graduate	187 (38.6%)	52 (26.0%)	
Some college	105 (21.6%)	46 (23.0%)	
High/Tech school	136 (28.0%)	70 (35.0%)	
<High school	57 (11.8%)	32 (16.0%)	
Income level, *n* (%)			0.001
<$30,000	132 (27.2%)	76 (38.0%)	
$30,000–$74,999	123 (25.4%)	48 (24.0%)	
$75,000–$99,999	59 (12.2%)	9 (4.5%)	
≥$100,000	62 (12.8%)	15 (7.5%)	
Prefer not to answer/do not know	109 (22.5%)	52 (26.0%)	
Gestational diabetes, *n* (%)			0.105
Yes	28 (5.8%)	5 (2.5%)	
No	457 (94.2%)	195 (97.5%)	
Preeclampsia, *n* (%)			0.018
Yes	15 (3.1%)	15 (7.5%)	
No	470 (96.9%)	185 (92.5%)	
Paternal Characteristics			
Asthma, *n* (%)			0.992
Yes	114 (23.5%)	46 (23.1%)	
No	371 (76.5%)	153 (76.9%) ^c^	
Characteristics at Birth			
Mode of delivery, *n* (%)			0.697
Cesarean	139 (28.7%)	61 (30.5%)	
Vaginal	346 (71.3%)	139 (69.5%)	
Delivery <37 weeks, *n* (%)			<0.001
Yes	24 (4.9%)	26 (13.0%)	
No	461 (95.1%)	174 (87.0%)	
Child sex, *n* (%)			0.007
Female	248 (51.1%)	79 (39.5%)	
Male	237 (48.9%)	121 (60.5%)	
Birth weight, kg, mean (SD)	3.3 (0.5)	3.2 (0.6)	0.034
Birth length, cm, mean (SD)	50.9 (2.7)	50.6 (3.1)	0.176

^a^ 57 missing; ^b^ 33 missing; ^c^ 1 missing; Abbreviations: SD, standard deviation; BMI, body mass index.

## Data Availability

The data presented in this study are available upon request.

## Supplementary Materials

The following are available online at https://www.mdpi.com/2218-1989/11/2/65/s1, Figure S1: Study sample flowchart, Figure S2: Schematic of main analyses results for the relation between maternal pregnancy metabolomes at two time points and child asthma or recurrent wheeze status by age three, Table S1: Maternal baseline (10–18 weeks gestation) metabolites with *p*-values < 0.05 for their associations with child asthma/recurrent wheeze status by age 3 in VDAART (sorted by *p*-values), Table S2: Maternal third trimester (32–38 weeks gestation) metabolites with *p*-values < 0.05 for their associations with child asthma/recurrent wheeze status by age 3 in VDAART (sorted by *p*-values), Table S3: Maternal baseline (10–18 weeks gestation) metabolites with *p*-values < 0.05 for their associations with child asthma/recurrent wheeze status by age 3 in girls in VDAART (sorted by *p*-values), Table S4: Maternal baseline (10–18 weeks gestation) metabolites with *p*-values < 0.05 for their associations with child asthma/recurrent wheeze status by age 3 in boys in VDAART (sorted by *p*-values), Table S5: Maternal third trimester (32–38 weeks gestation) metabolites with *p*-values < 0.05 for their associations with child asthma/recurrent wheeze status by age 3 in girls in VDAART (sorted by *p*-values), Table S6: Maternal third trimester (32–38 weeks gestation) metabolites with *p*-values < 0.05 for their associations with child asthma/recurrent wheeze status by age 3 in boys in VDAART (sorted by *p*-values), Table S7: Maternal baseline (10–18 weeks gestation) metabolites with *p*-values < 0.05 for their interaction with child sex in association with child asthma/recurrent wheeze status by age 3 in VDAART (sorted by interaction term *p*-values), Table S8: Maternal third trimester (32–38 weeks gestation) metabolites with *p*-values < 0.05 for their interaction with child sex in association with child asthma/recurrent wheeze status by age 3 in VDAART (sorted by interaction term *p*-values).
