# Supplementary material for: Maternal Metabolome in Pregnancy and Childhood Asthma or Recurrent Wheeze in the Vitamin D Antenatal Asthma Reduction Trial
Source: Metabolites. 2021 Jan 23;11(2):65. doi: 10.3390/metabo11020065 (PMC7910853; doi:10.3390/metabo11020065)
Supplement: Supplementary file 1 [file metabolites-11-00065-s001.pdf]

Title: Maternal Metabolome in Pregnancy and Childhood Asthma or Recurrent Wheeze in the Vitamin D Antenatal Asthma Reduction Trial

Author Names: Mengna Huang, Rachel S. Kelly, Su H. Chu, Priyadarshini Kachroo, Gözde Gürdeniz, Bo L. Chawes, Hans Bisgaard, Scott T. Weiss, Jessica Lasky-Su

Supplementary Tables and Figures

Contents

Supplementary Figure S1. Study sample flowchart 2

Supplementary Figure S2. Schematic of main analyses results for the relation between maternal pregnancy metabolomes at two time points and child asthma or recurrent wheeze status by age three 3

Supplementary Table S1. Maternal baseline (10–18 weeks gestation) metabolites with *p*-values < 0.05 for their associations with child asthma/recurrent wheeze status by age 3 in VDAART (sorted by *p*-values) 4

Supplementary Table S2. Maternal third trimester (32–38 weeks gestation) metabolites with *p*-values < 0.05 for their associations with child asthma/recurrent wheeze status by age 3 in VDAART (sorted by *p*-values) 5

Supplementary Table S3. Maternal baseline (10–18 weeks gestation) metabolites with *p*-values < 0.05 for their associations with child asthma/recurrent wheeze status by age 3 in girls in VDAART (sorted by *p*-values) 8

Supplementary Table S4. Maternal baseline (10–18 weeks gestation) metabolites with *p*-values < 0.05 for their associations with child asthma/recurrent wheeze status by age 3 in boys in VDAART (sorted by *p*-values) 10

Supplementary Table S5. Maternal third trimester (32–38 weeks gestation) metabolites with *p*-values < 0.05 for their associations with child asthma/recurrent wheeze status by age 3 in girls in VDAART (sorted by *p*-values) 12

Supplementary Table S6. Maternal third trimester (32–38 weeks gestation) metabolites with *p*-values < 0.05 for their associations with child asthma/recurrent wheeze status by age 3 in boys in VDAART (sorted by *p*-values) 15

Supplementary Table S7. Maternal baseline (10–18 weeks gestation) metabolites with *p*-values < 0.05 for their interaction with child sex in association with child asthma/recurrent wheeze status by age 3 in VDAART (sorted by interaction term *p*-values) 18

Supplementary Table S8. Maternal third trimester (32–38 weeks gestation) metabolites with *p*-values < 0.05 for their interaction with child sex in association with child asthma/recurrent wheeze status by age 3 in VDAART (sorted by interaction term *p*-values) 20

Metabolomic profiling in the Vitamin D Antenatal Asthma Reduction Trial (VDAART) 22

Supplementary Figure S1. Study sample flowchart

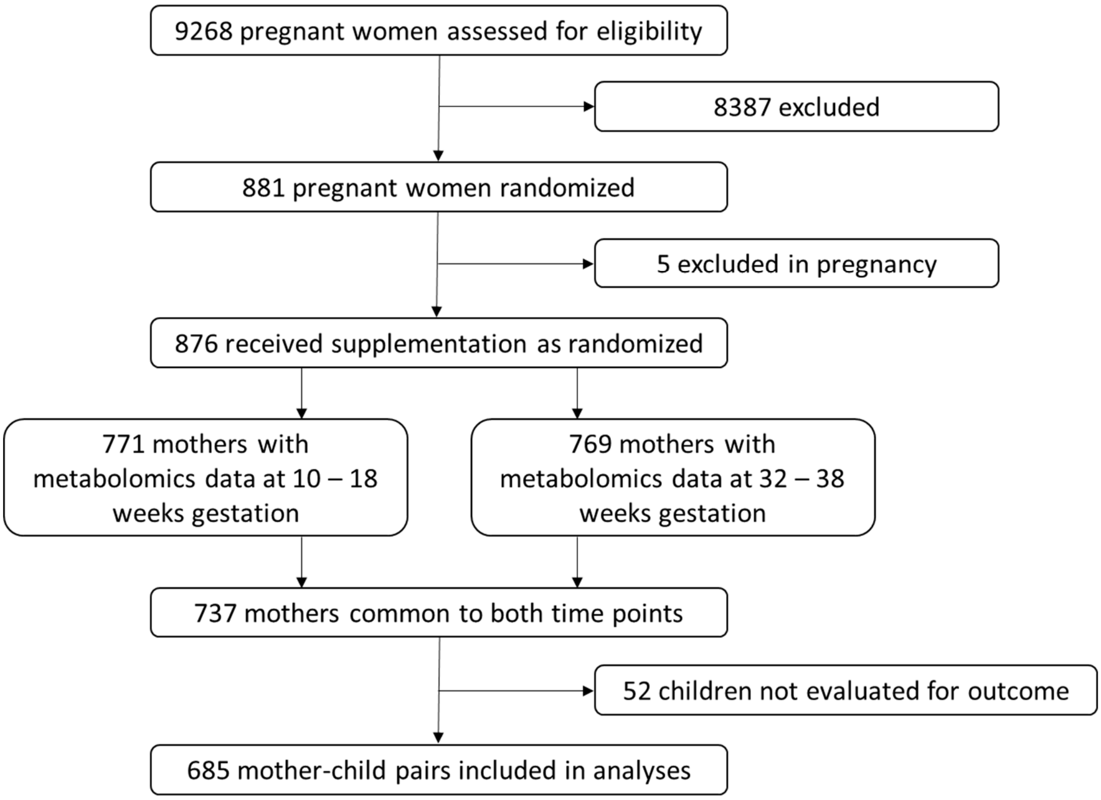

Supplementary Figure S2. Schematic of main analyses results for the relation between maternal pregnancy metabolomes at two time points and child asthma or recurrent wheeze status by age three

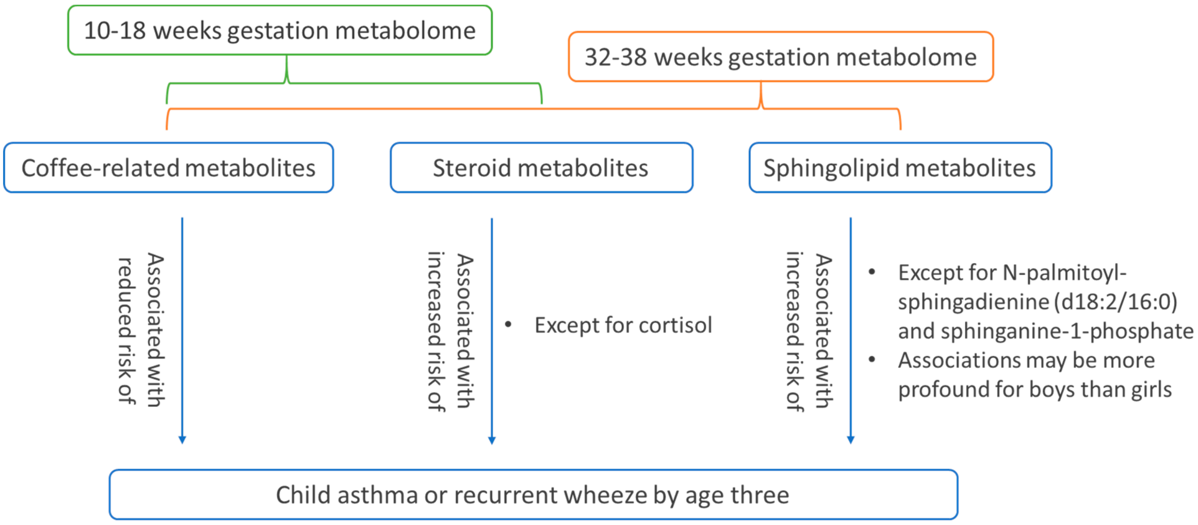

Supplementary Table S1. Maternal baseline (10–18 weeks gestation) metabolites with *p*-values < 0.05 for their associations with child asthma/recurrent wheeze status by age 3 in VDAART (sorted by *p*-values)

Metabolite	Sub-pathway	Super-pathway	Beta estimate	p-value	Odds ratio	95% CI lower bound	95% CI upper bound
2-aminoadipate	Lysine Metabolism	Amino Acid	0.61	5.24E-05	1.84	1.37	2.47
pregnenetriol sulfate*	Pregnenolone Steroids	Lipid	0.47	2.27E-02	1.60	1.07	2.40
pregnenolone sulfate	Pregnenolone Steroids	Lipid	0.48	2.40E-02	1.62	1.07	2.45
1-linoleoyl-2-arachidonoyl-GPC (18:2/20:4n6)*	Phosphatidylcholine (PC)	Lipid	0.58	2.68E-02	1.79	1.07	2.99
theophylline	Xanthine Metabolism	Xenobiotics	-0.24	2.72E-02	0.79	0.64	0.97
quinate	Food Component/Plant	Xenobiotics	-0.24	2.77E-02	0.79	0.64	0.97
thyroxine	Tyrosine Metabolism	Amino Acid	0.74	3.21E-02	2.10	1.07	4.12
sphingomyelin (d18:2/24:1, d18:1/24:2)*	Sphingomyelins	Lipid	0.70	3.25E-02	2.01	1.06	3.79
propionylglycine (C3)	Fatty Acid Metabolism (also BCAA Metabolism)	Lipid	0.37	3.42E-02	1.45	1.03	2.04
3-hydroxypyridine sulfate	Chemical	Xenobiotics	-0.25	3.57E-02	0.78	0.61	0.98
pregnenediol sulfate (C21H34O5S)*	Pregnenolone Steroids	Lipid	0.46	3.75E-02	1.58	1.03	2.43
1-palmitoyl-2-arachidonoyl-GPI (16:0/20:4)*	Phosphatidylinositol (PI)	Lipid	0.47	3.85E-02	1.60	1.03	2.51
caffeine	Xanthine Metabolism	Xenobiotics	-0.20	3.88E-02	0.82	0.68	0.99
1-(1-enyl-palmitoyl)-2-linoleoyl-GPC (P-16:0/18:2)*	Plasmalogen	Lipid	0.56	4.19E-02	1.75	1.02	2.98
propyl 4-hydroxybenzoate sulfate	Benzoate Metabolism	Xenobiotics	-0.20	4.30E-02	0.82	0.67	0.99
dehydroepiandrosterone sulfate (DHEA-S)	Androgenic Steroids	Lipid	0.37	4.63E-02	1.44	1.01	2.07
androstenediol (3alpha, 17alpha) monosulfate	Androgenic Steroids	Lipid	0.32	4.68E-02	1.38	1.00	1.89
beta-cryptoxanthin	Vitamin A Metabolism	Cofactors and Vitamins	0.34	4.76E-02	1.41	1.00	1.98
carotene diol	Vitamin A Metabolism	Cofactors and Vitamins	0.43	4.94E-02	1.54	1.00	2.38

*: indicates a compound that has not been confirmed based on a standard, but we are confident in its identity.

Abbreviations: BCAA, branched chain amino acid; VDAART, Vitamin D Antenatal Asthma Reduction Trial.

Supplementary Table S2. Maternal third trimester (32–38 weeks gestation) metabolites with *p*-values < 0.05 for their associations with child asthma/recurrent wheeze status by age 3 in VDAART (sorted by *p*-values)

Metabolite	Sub-pathway	Super-pathway	Beta estimate	p-value	Odds ratio	95% CI lower bound	95% CI upper bound
N-palmitoylglycine	Fatty Acid Metabolism (Acyl Glycine)	Lipid	0.70	2.10E-04	2.01	1.39	2.91
sulfate*	Chemical	Xenobiotics	1.06	1.02E-03	2.87	1.53	5.39
palmitoyl sphingomyelin (d18:1/16:0)	Sphingomyelins	Lipid	1.22	1.59E-03	3.38	1.59	7.18
sphingomyelin (d18:2/24:2)*	Sphingomyelins	Lipid	0.89	3.39E-03	2.43	1.34	4.40
3-hydroxyhexanoate	Fatty Acid, Monohydroxy	Lipid	0.57	3.65E-03	1.77	1.20	2.59
hexanoylglutamine	Fatty Acid Metabolism (Acyl Glutamine)	Lipid	0.36	4.24E-03	1.43	1.12	1.82
diacylglycerol (16:1/18:2 [2], 16:0/18:3 [1])*	Diacylglycerol	Lipid	-0.34	4.43E-03	0.71	0.56	0.90
mannose	Fructose, Mannose and Galactose Metabolism	Carbohydrate	0.31	5.04E-03	1.36	1.10	1.68
pregnenetriol disulfate*	Pregnenolone Steroids	Lipid	0.51	5.37E-03	1.67	1.16	2.40
2-hydroxystearate	Fatty Acid, Monohydroxy	Lipid	0.61	8.59E-03	1.83	1.17	2.88
sphingomyelin (d18:2/16:0, d18:1/16:1)*	Sphingomyelins	Lipid	0.86	8.90E-03	2.37	1.24	4.53
O-sulfo-L-tyrosine	Chemical	Xenobiotics	0.61	1.09E-02	1.84	1.15	2.96
cis-4-decenoate (10:1n6)*	Medium Chain Fatty Acid	Lipid	0.43	1.15E-02	1.54	1.10	2.16
2-hydroxypalmitate	Fatty Acid, Monohydroxy	Lipid	0.59	1.16E-02	1.80	1.14	2.84
lignoceroyl sphingomyelin (d18:1/24:0)	Sphingomyelins	Lipid	0.72	1.19E-02	2.06	1.17	3.61
16a-hydroxy DHEA 3-sulfate	Androgenic Steroids	Lipid	0.48	1.25E-02	1.62	1.11	2.36
octadecanedioate (C18)	Fatty Acid, Dicarboxylate	Lipid	0.48	1.28E-02	1.62	1.11	2.36
gulonate*	Ascorbate and Aldarate Metabolism	Cofactors and Vitamins	-0.27	1.34E-02	0.76	0.61	0.95
cortisol	Corticosteroids	Lipid	-0.55	1.38E-02	0.58	0.37	0.89
linoleate (18:2n6)	Long Chain Polyunsaturated Fatty Acid (n3 and n6)	Lipid	0.41	1.44E-02	1.51	1.09	2.10
hydroxypalmitoyl sphingomyelin (d18:1/16:0(OH))	Sphingomyelins	Lipid	0.68	1.55E-02	1.98	1.14	3.43

Metabolite	Sub-pathway	Super-pathway	Beta estimate	p-value	Odds ratio	95% CI lower bound	95% CI upper bound
3-phosphoglycerate	Glycolysis, Gluconeogenesis, and Pyruvate Metabolism	Carbohydrate	-0.42	1.68E-02	0.66	0.47	0.93
glucose	Glycolysis, Gluconeogenesis, and Pyruvate Metabolism	Carbohydrate	0.26	1.73E-02	1.30	1.05	1.60
oleate/vaccenate (18:1)	Long Chain Monounsaturated Fatty Acid	Lipid	0.39	1.84E-02	1.48	1.07	2.05
1-linoleoyl-GPI* (18:2)*	Lysophospholipid	Lipid	0.42	2.13E-02	1.53	1.06	2.19
thyroxine	Tyrosine Metabolism	Amino Acid	0.65	2.26E-02	1.91	1.10	3.33
2-hydroxybehenate	Fatty Acid, Monohydroxy	Lipid	0.53	2.33E-02	1.71	1.08	2.71
pseudouridine	Pyrimidine Metabolism, Uracil containing	Nucleotide	0.75	2.37E-02	2.11	1.10	4.03
cys-gly, oxidized	Glutathione Metabolism	Amino Acid	-0.41	2.56E-02	0.67	0.47	0.95
sphingomyelin (d18:2/24:1, d18:1/24:2)*	Sphingomyelins	Lipid	0.75	2.57E-02	2.12	1.10	4.09
dihomolinoleate (20:2n6)	Long Chain Polyunsaturated Fatty Acid (n3 and n6)	Lipid	0.41	2.65E-02	1.51	1.05	2.18
N-acetylaspartate (NAA)	Alanine and Aspartate Metabolism	Amino Acid	0.50	2.78E-02	1.65	1.06	2.57
pregnen-diol disulfate*	Pregnenolone Steroids	Lipid	0.46	2.89E-02	1.59	1.05	2.41
sphinganine-1-phosphate	Sphingolipid Synthesis	Lipid	-0.46	2.89E-02	0.63	0.42	0.95
methylsuccinate	Leucine, Isoleucine and Valine Metabolism	Amino Acid	-0.37	2.96E-02	0.69	0.49	0.96
1-(1-enyl-palmitoyl)-2-palmitoleoyl-GPC (P-16:0/16:1)*	Plasmalogen	Lipid	0.59	2.98E-02	1.81	1.06	3.10
2-oxoarginine*	Urea cycle; Arginine and Proline Metabolism	Amino Acid	-0.40	3.04E-02	0.67	0.47	0.96
3-methoxytyrosine	Tyrosine Metabolism	Amino Acid	-0.58	3.06E-02	0.56	0.33	0.95
5-methylthioadenosine (MTA)	Polyamine Metabolism	Amino Acid	0.35	3.10E-02	1.43	1.03	1.97
5,6-dihydrouridine	Pyrimidine Metabolism, Uracil containing	Nucleotide	0.67	3.10E-02	1.95	1.06	3.57
sphingomyelin (d18:1/24:1, d18:2/24:0)*	Sphingomyelins	Lipid	0.71	3.20E-02	2.03	1.06	3.87
oxalate (ethanedioate)	Ascorbate and Aldarate Metabolism	Cofactors and Vitamins	0.55	3.21E-02	1.74	1.05	2.88

Metabolite	Sub-pathway	Super-pathway	Beta estimate	p-value	Odds ratio	95% CI lower bound	95% CI upper bound
eicosanedioate (C20-DC)	Fatty Acid, Dicarboxylate	Lipid	0.42	3.29E-02	1.53	1.04	2.25
salicylate	Drug - Topical Agents	Xenobiotics	-0.32	3.32E-02	0.73	0.54	0.98
1-methylnicotinamide	Nicotinate and Nicotinamide Metabolism	Cofactors and Vitamins	-0.40	3.49E-02	0.67	0.46	0.97
stearate (18:0)	Long Chain Saturated Fatty Acid	Lipid	0.45	3.68E-02	1.57	1.03	2.41
2-aminophenol sulfate	Food Component/Plant	Xenobiotics	-0.29	3.68E-02	0.75	0.57	0.98
N-palmitoyl-sphingadienine (d18:2/16:0)*	Ceramides	Lipid	-0.30	3.72E-02	0.74	0.56	0.98
1-arachidonoyl-GPI* (20:4)*	Lysophospholipid	Lipid	0.42	3.76E-02	1.52	1.02	2.27
choline	Phospholipid Metabolism	Lipid	-0.71	3.95E-02	0.49	0.25	0.97
dodecadienoate (12:2)*	Fatty Acid, Dicarboxylate	Lipid	0.36	4.05E-02	1.43	1.02	2.02
pipecolate	Lysine Metabolism	Amino Acid	-0.42	4.10E-02	0.66	0.44	0.98
bilirubin (E,E)*	Hemoglobin and Porphyrin Metabolism	Cofactors and Vitamins	0.31	4.13E-02	1.36	1.01	1.83
1-linoleoyl-GPC (18:2)	Lysophospholipid	Lipid	0.44	4.23E-02	1.55	1.02	2.37
trigonelline (N'-methylnicotinate)	Nicotinate and Nicotinamide Metabolism	Cofactors and Vitamins	-0.33	4.26E-02	0.72	0.53	0.99
sphingomyelin (d18:1/20:2, d18:2/20:1, d16:1/22:2)*	Sphingomyelins	Lipid	0.53	4.31E-02	1.70	1.02	2.84
cysteinylglycine disulfide*	Glutathione Metabolism	Amino Acid	-0.51	4.32E-02	0.60	0.37	0.98
succinylcarnitine (C4-DC)	TCA Cycle	Energy	-0.53	4.51E-02	0.59	0.35	0.99
quinate	Food Component/Plant	Xenobiotics	-0.21	4.57E-02	0.81	0.65	1.00
3-hydroxypyridine sulfate	Chemical	Xenobiotics	-0.23	4.62E-02	0.80	0.64	1.00
3-hydroxybutyrate (BHBA)	Ketone Bodies	Lipid	0.28	4.72E-02	1.32	1.00	1.75
AMP	Purine Metabolism, Adenine containing	Nucleotide	-0.38	4.97E-02	0.69	0.47	1.00

*: indicates a compound that has not been confirmed based on a standard, but we are confident in its identity.

Abbreviations: VDAART, Vitamin D Antenatal Asthma Reduction Trial.

Supplementary Table S3. Maternal baseline (10–18 weeks gestation) metabolites with *p*-values < 0.05 for their associations with child asthma/recurrent wheeze status by age 3 in girls in VDAART (sorted by *p*-values)

**Metabolite**	**Sub-pathway**	**Super-pathway**	**Beta estimate**	**p-value**	**Odds ratio**	**95% CI lower bound**	**95% CI upper bound**
2-aminoadipate	Lysine Metabolism	Amino Acid	0.81	1.94E-03	2.25	1.35	3.75
theobromine	Xanthine Metabolism	Xenobiotics	-0.41	2.44E-03	0.66	0.51	0.87
1-(1-enyl-palmitoyl)-2-linoleoyl-GPC (P-16:0/18:2)*	Plasmalogen	Lipid	1.34	3.46E-03	3.82	1.56	9.38
isoursodeoxycholate	Secondary Bile Acid Metabolism	Lipid	0.59	6.17E-03	1.81	1.18	2.76
3-methylxanthine	Xanthine Metabolism	Xenobiotics	-0.56	7.45E-03	0.57	0.38	0.86
theophylline	Xanthine Metabolism	Xenobiotics	-0.46	7.87E-03	0.63	0.45	0.89
glycolithocholate sulfate*	Secondary Bile Acid Metabolism	Lipid	0.62	1.44E-02	1.86	1.13	3.06
pregnenolone sulfate	Pregnenolone Steroids	Lipid	0.83	1.45E-02	2.30	1.18	4.48
glyco-beta-muricholate	Primary Bile Acid Metabolism	Lipid	0.50	1.59E-02	1.65	1.10	2.48
hexadecenedioate (C16:1-DC)*	Fatty Acid, Dicarboxylate	Lipid	0.68	1.61E-02	1.97	1.13	3.43
octadecenedioate (C18:1-DC)*	Fatty Acid, Dicarboxylate	Lipid	0.68	1.76E-02	1.98	1.13	3.49
thyroxine	Tyrosine Metabolism	Amino Acid	1.37	1.86E-02	3.92	1.26	12.25
caffeine	Xanthine Metabolism	Xenobiotics	-0.37	1.86E-02	0.69	0.51	0.94
androstenediol (3alpha, 17alpha) monosulfate (2)	Androgenic Steroids	Lipid	0.65	1.87E-02	1.91	1.11	3.28
1,2-dilinoleoyl-GPC (18:2/18:2)	Phosphatidylcholine (PC)	Lipid	0.88	2.02E-02	2.40	1.15	5.02
17alpha-hydroxypregnanolone glucuronide	Pregnenolone Steroids	Lipid	-0.62	2.10E-02	0.54	0.32	0.91
p-cresol sulfate	Benzoate Metabolism	Xenobiotics	0.62	2.12E-02	1.86	1.10	3.15
1-palmitoylglycerol (16:0)	Monoacylglycerol	Lipid	-0.45	2.19E-02	0.64	0.44	0.94
1-(1-enyl-palmitoyl)-GPC (P-16:0)*	Lysoplasmalogen	Lipid	0.76	2.31E-02	2.15	1.11	4.15
N-oleoylserine	Endocannabinoid	Lipid	0.72	2.98E-02	2.06	1.07	3.95
3-amino-2-piperidone	Urea cycle; Arginine and Proline Metabolism	Amino Acid	0.68	3.00E-02	1.98	1.07	3.66
p-cresol glucuronide*	Tyrosine Metabolism	Amino Acid	0.40	3.02E-02	1.50	1.04	2.16

Metabolite	Sub-pathway	Super-pathway	Beta estimate	p-value	Odds ratio	95% CI lower bound	95% CI upper bound
thioproline	Chemical	Xenobiotics	-0.95	3.72E-02	0.39	0.16	0.95
hydroxy-N6,N6,N6-trimethyllysine*	Lysine Metabolism	Amino Acid	-0.57	3.81E-02	0.57	0.33	0.97
homocitrulline	Urea cycle; Arginine and Proline Metabolism	Amino Acid	0.59	3.85E-02	1.80	1.03	3.15
6-oxopiperidine-2-carboxylate	Lysine Metabolism	Amino Acid	0.71	3.91E-02	2.03	1.04	3.96
7-methylguanine	Purine Metabolism, Guanine containing	Nucleotide	-0.67	4.31E-02	0.51	0.27	0.98
quinate	Food Component/Plant	Xenobiotics	-0.34	4.32E-02	0.71	0.51	0.99
3beta-hydroxy-5-cholestenoate	Sterol	Lipid	0.76	4.38E-02	2.13	1.02	4.44
1-(1-enyl-stearoyl)-2-linoleoyl-GPE (P-18:0/18:2)*	Plasmalogen	Lipid	0.67	4.59E-02	1.96	1.01	3.79
1,7-dimethylurate	Xanthine Metabolism	Xenobiotics	-0.35	4.59E-02	0.70	0.50	0.99
deoxycholate	Secondary Bile Acid Metabolism	Lipid	0.44	4.70E-02	1.56	1.01	2.41

*: indicates a compound that has not been confirmed based on a standard, but we are confident in its identity.

Abbreviations: VDAART, Vitamin D Antenatal Asthma Reduction Trial.

Supplementary Table S4. Maternal baseline (10–18 weeks gestation) metabolites with *p*-values < 0.05 for their associations with child asthma/recurrent wheeze status by age 3 in boys in VDAART (sorted by *p*-values)

Metabolite	Sub-pathway	Super-pathway	Beta estimate	p-value	Odds ratio	95% CI lower bound	95% CI upper bound
12,13-DiHOME	Fatty Acid, Dihydroxy	Lipid	-0.57	4.48E-03	0.56	0.38	0.84
2-aminoadipate	Lysine Metabolism	Amino Acid	0.50	9.03E-03	1.65	1.13	2.40
kynurenine	Tryptophan Metabolism	Amino Acid	1.06	1.07E-02	2.89	1.28	6.52
3-hydroxypyridine sulfate	Chemical	Xenobiotics	-0.40	1.61E-02	0.67	0.49	0.93
guaiacol sulfate	Benzoate Metabolism	Xenobiotics	-0.57	1.69E-02	0.57	0.36	0.90
thioproline	Chemical	Xenobiotics	0.96	1.93E-02	2.61	1.17	5.83
catechol sulfate	Benzoate Metabolism	Xenobiotics	-0.59	1.94E-02	0.56	0.34	0.91
3-methyl catechol sulfate (1)	Benzoate Metabolism	Xenobiotics	-0.33	2.07E-02	0.72	0.55	0.95
9,10-DiHOME	Fatty Acid, Dihydroxy	Lipid	-0.44	2.21E-02	0.64	0.44	0.94
cysteine-glutathione disulfide	Glutathione Metabolism	Amino Acid	0.38	2.67E-02	1.46	1.04	2.04
maltotriose	Glycogen Metabolism	Carbohydrate	0.29	3.09E-02	1.34	1.03	1.74
pseudouridine	Pyrimidine Metabolism, Uracil containing	Nucleotide	1.00	3.12E-02	2.73	1.09	6.78
gamma-glutamylglutamine	Gamma-glutamyl Amino Acid	Peptide	0.61	3.38E-02	1.84	1.05	3.22
maltose	Glycogen Metabolism	Carbohydrate	0.33	3.42E-02	1.40	1.03	1.91
hyocholate	Secondary Bile Acid Metabolism	Lipid	0.33	3.56E-02	1.40	1.02	1.91
1-stearoyl-2-oleoyl-GPS (18:0/18:1)	Phosphatidylserine (PS)	Lipid	0.50	3.66E-02	1.64	1.03	2.62
trigonelline (N'-methylnicotinate)	Nicotinate and Nicotinamide Metabolism	Cofactors and Vitamins	-0.42	4.26E-02	0.66	0.44	0.99
glutarate (C5-DC)	Fatty Acid, Dicarboxylate	Lipid	0.34	4.28E-02	1.41	1.01	1.96
uridine	Pyrimidine Metabolism, Uracil containing	Nucleotide	0.83	4.56E-02	2.28	1.02	5.13
1-palmitoyl-2-arachidonoyl-GPI (16:0/20:4)*	Phosphatidylinositol (PI)	Lipid	0.61	4.64E-02	1.85	1.01	3.38
hydroxyasparagine	Alanine and Aspartate Metabolism	Amino Acid	0.80	4.70E-02	2.23	1.01	4.92

Metabolite	Sub-pathway	Super-pathway	Beta estimate	p-value	Odds ratio	95% CI lower bound	95% CI upper bound
gamma-glutamyltryptophan	Gamma-glutamyl Amino Acid	Peptide	0.45	4.83E-02	1.56	1.00	2.44
sphingomyelin (d18:2/24:1, d18:1/24:2)*	Sphingomyelins	Lipid	0.88	4.88E-02	2.42	1.00	5.82

*: indicates a compound that has not been confirmed based on a standard, but we are confident in its identity.

Abbreviations: VDAART, Vitamin D Antenatal Asthma Reduction Trial.

Supplementary Table S5. Maternal third trimester (32–38 weeks gestation) metabolites with *p*-values < 0.05 for their associations with child asthma/recurrent wheeze status by age 3 in girls in VDAART (sorted by *p*-values)

Metabolite	Sub-pathway	Super-pathway	Beta estimate	p-value	Odds ratio	95% CI lower bound	95% CI upper bound
salicylate	Drug - Topical Agents	Xenobiotics	-1.04	3.38E-04	0.35	0.20	0.62
2-hydroxyhippurate (salicylurate)	Benzoate Metabolism	Xenobiotics	-0.77	4.28E-04	0.46	0.30	0.71
N-acetyl-isoputreanine	Polyamine Metabolism	Amino Acid	-1.18	4.35E-03	0.31	0.14	0.69
N2,N2-dimethylguanosine	Purine Metabolism, Guanine containing	Nucleotide	-1.34	4.83E-03	0.26	0.10	0.67
hexadecenedioate (C16:1-DC)*	Fatty Acid, Dicarboxylate	Lipid	0.91	5.44E-03	2.48	1.31	4.72
1-stearoyl-2-docosahexaenoyl-GPE (18:0/22:6)*	Phosphatidylethanolamine (PE)	Lipid	-1.09	5.67E-03	0.34	0.16	0.73
cysteinylglycine disulfide*	Glutathione Metabolism	Amino Acid	-1.07	6.87E-03	0.34	0.16	0.75
1,7-dimethylurate	Xanthine Metabolism	Xenobiotics	-0.49	8.00E-03	0.61	0.43	0.88
octadecadienedioate (C18:2-DC)*	Fatty Acid, Dicarboxylate	Lipid	0.74	8.53E-03	2.10	1.21	3.65
2R,3R-dihydroxybutyrate	Fatty Acid, Dihydroxy	Lipid	-0.97	9.43E-03	0.38	0.18	0.79
cys-gly, oxidized	Glutathione Metabolism	Amino Acid	-0.74	1.17E-02	0.48	0.27	0.85
citramalate	Glutamate Metabolism	Amino Acid	-0.47	1.18E-02	0.62	0.43	0.90
caffeine	Xanthine Metabolism	Xenobiotics	-0.43	1.20E-02	0.65	0.47	0.91
beta-cryptoxanthin	Vitamin A Metabolism	Cofactors and Vitamins	0.74	1.38E-02	2.10	1.16	3.79
thyroxine	Tyrosine Metabolism	Amino Acid	1.10	1.47E-02	3.01	1.24	7.30
oxalate (ethanedioate)	Ascorbate and Aldarate Metabolism	Cofactors and Vitamins	1.09	1.67E-02	2.97	1.22	7.25
AMP	Purine Metabolism, Adenine containing	Nucleotide	-0.72	1.76E-02	0.48	0.27	0.88
succinylcarnitine (C4-DC)	TCA Cycle	Energy	-0.92	1.81E-02	0.40	0.19	0.85
7-methylguanine	Purine Metabolism, Guanine containing	Nucleotide	-1.21	1.83E-02	0.30	0.11	0.82
palmitoyl sphingomyelin (d18:1/16:0)	Sphingomyelins	Lipid	1.48	1.90E-02	4.40	1.28	15.15
1-palmitoyl-2-docosahexaenoyl-GPE (16:0/22:6)*	Phosphatidylethanolamine (PE)	Lipid	-0.95	1.93E-02	0.39	0.17	0.86
eicosanedioate (C20-DC)	Fatty Acid, Dicarboxylate	Lipid	0.71	2.00E-02	2.04	1.12	3.73

Metabolite	Sub-pathway	Super-pathway	Beta estimate	p-value	Odds ratio	95% CI lower bound	95% CI upper bound
theophylline	Xanthine Metabolism	Xenobiotics	-0.42	2.11E-02	0.65	0.46	0.94
mannose	Fructose, Mannose and Galactose Metabolism	Carbohydrate	0.40	2.34E-02	1.49	1.06	2.09
C-glycosyltryptophan	Tryptophan Metabolism	Amino Acid	-1.08	2.39E-02	0.34	0.13	0.87
2-hydroxybehenate	Fatty Acid, Monohydroxy	Lipid	0.85	2.50E-02	2.33	1.11	4.88
N-acetylcarnosine	Histidine Metabolism	Amino Acid	-0.49	2.50E-02	0.61	0.40	0.94
cortisol	Corticosteroids	Lipid	-0.80	2.51E-02	0.45	0.22	0.90
tetradecanedioate (C14)	Fatty Acid, Dicarboxylate	Lipid	0.72	2.59E-02	2.04	1.09	3.84
N-palmitoylglycine	Fatty Acid Metabolism (Acyl Glycine)	Lipid	0.64	2.67E-02	1.90	1.08	3.35
3-hydroxybutyrate (BHBA)	Ketone Bodies	Lipid	0.47	2.72E-02	1.61	1.05	2.45
ADP	Purine Metabolism, Adenine containing	Nucleotide	-0.48	2.76E-02	0.62	0.40	0.95
N1-methylinosine	Purine Metabolism, (Hypo)Xanthine/Inosine containing	Nucleotide	-0.81	2.78E-02	0.45	0.22	0.92
sulfate*	Chemical	Xenobiotics	1.18	3.18E-02	3.27	1.11	9.64
glucose	Glycolysis, Gluconeogenesis, and Pyruvate Metabolism	Carbohydrate	0.38	3.58E-02	1.46	1.03	2.08
cortolone glucuronide (1)	Corticosteroids	Lipid	-0.54	3.87E-02	0.58	0.35	0.97
1,2-dilinoleoyl-GPC (18:2/18:2)	Phosphatidylcholine (PC)	Lipid	0.92	3.87E-02	2.52	1.05	6.06
sphingosine	Sphingosines	Lipid	-0.72	4.02E-02	0.48	0.24	0.97
glutamate	Glutamate Metabolism	Amino Acid	-0.84	4.05E-02	0.43	0.19	0.96
lignoceroyl sphingomyelin (d18:1/24:0)	Sphingomyelins	Lipid	0.94	4.06E-02	2.55	1.04	6.25
hexanoylglutamine	Fatty Acid Metabolism (Acyl Glutamine)	Lipid	0.41	4.08E-02	1.51	1.02	2.24
orotidine	Pyrimidine Metabolism, Orotate containing	Nucleotide	-0.80	4.12E-02	0.45	0.21	0.97
phosphoethanolamine (PE)	Phospholipid Metabolism	Lipid	-0.56	4.12E-02	0.57	0.33	0.98
creatinine	Creatine Metabolism	Amino Acid	-1.28	4.18E-02	0.28	0.08	0.95
hexadecanedioate (C16)	Fatty Acid, Dicarboxylate	Lipid	0.67	4.20E-02	1.95	1.02	3.69

Metabolite	Sub-pathway	Super-pathway	Beta estimate	p-value	Odds ratio	95% CI lower bound	95% CI upper bound
kynurenine	Tryptophan Metabolism	Amino Acid	-0.89	4.50E-02	0.41	0.17	0.98
dihomo-linolenoylcarnitine (C20:3n3 or 6)*	Fatty Acid Metabolism (Acyl Carnitine, Polyunsaturated)	Lipid	-0.52	4.51E-02	0.60	0.36	0.99
2-hydroxylaurate	Fatty Acid, Monohydroxy	Lipid	0.84	4.68E-02	2.31	1.01	5.29
2-hydroxyphenylacetate	Phenylalanine Metabolism	Amino Acid	0.53	4.74E-02	1.70	1.01	2.86
carotene diol (2)	Vitamin A Metabolism	Cofactors and Vitamins	0.75	4.83E-02	2.11	1.01	4.44
2,3-dihydroxyisovalerate	Food Component/Plant	Xenobiotics	0.35	4.93E-02	1.42	1.00	2.00
cortisone	Corticosteroids	Lipid	-0.71	4.94E-02	0.49	0.24	1.00

*: indicates a compound that has not been confirmed based on a standard, but we are confident in its identity.

Abbreviations: VDAART, Vitamin D Antenatal Asthma Reduction Trial.

Supplementary Table S6. Maternal third trimester (32–38 weeks gestation) metabolites with *p*-values < 0.05 for their associations with child asthma/recurrent wheeze status by age 3 in boys in VDAART (sorted by *p*-values)

Metabolite	Sub-pathway	Super-pathway	Beta estimate	p-value	Odds ratio	95% CI lower bound	95% CI upper bound
sphingomyelin (d18:2/24:2)*	Sphingomyelins	Lipid	1.33	1.33E-03	3.77	1.68	8.48
5,6-dihydrouridine	Pyrimidine Metabolism, Uracil containing	Nucleotide	1.36	1.80E-03	3.91	1.66	9.21
diacylglycerol (16:1/18:2 [2], 16:0/18:3 [1])*	Diacylglycerol	Lipid	-0.49	2.35E-03	0.61	0.45	0.84
cis-4-decenoate (10:1n6)*	Medium Chain Fatty Acid	Lipid	0.73	2.44E-03	2.08	1.29	3.33
N6-carbamoylthreonyladenosine	Purine Metabolism, Adenine containing	Nucleotide	1.35	3.07E-03	3.84	1.58	9.38
N-palmitoylglycine	Fatty Acid Metabolism (Acyl Glycine)	Lipid	0.76	3.41E-03	2.13	1.28	3.53
quinolinate	Nicotinate and Nicotinamide Metabolism	Cofactors and Vitamins	0.92	3.88E-03	2.52	1.35	4.71
pseudouridine	Pyrimidine Metabolism, Uracil containing	Nucleotide	1.38	4.08E-03	3.98	1.55	10.20
sphingomyelin (d18:2/16:0, d18:1/16:1)*	Sphingomyelins	Lipid	1.32	4.23E-03	3.73	1.51	9.20
dodecanedioate (C12)	Fatty Acid, Dicarboxylate	Lipid	-0.66	4.41E-03	0.52	0.33	0.81
sphingomyelin (d18:1/24:1, d18:2/24:0)*	Sphingomyelins	Lipid	1.32	4.46E-03	3.75	1.51	9.32
O-sulfo-L-tyrosine	Chemical	Xenobiotics	0.97	4.58E-03	2.65	1.35	5.20
3-(3-amino-3-carboxypropyl)uridine*	Pyrimidine Metabolism, Uracil containing	Nucleotide	1.04	6.02E-03	2.83	1.35	5.94
2-hydroxypalmitate	Fatty Acid, Monohydroxy	Lipid	0.92	6.51E-03	2.50	1.29	4.84
sphingomyelin (d18:2/24:1, d18:1/24:2)*	Sphingomyelins	Lipid	1.24	7.50E-03	3.44	1.39	8.51
palmitoyl sphingomyelin (d18:1/16:0)	Sphingomyelins	Lipid	1.36	9.48E-03	3.90	1.39	10.88
3-hydroxyhexanoate	Fatty Acid, Monohydroxy	Lipid	0.72	9.70E-03	2.06	1.19	3.58
3-phosphoglycerate	Glycolysis, Gluconeogenesis, and Pyruvate Metabolism	Carbohydrate	-0.65	1.05E-02	0.52	0.32	0.86
aconitate [cis or trans]	TCA Cycle	Energy	1.37	1.05E-02	3.95	1.38	11.32

Metabolite	Sub-pathway	Super-pathway	Beta estimate	p-value	Odds ratio	95% CI lower bound	95% CI upper bound
2-stearoyl-GPE (18:0)*	Lysophospholipid	Lipid	0.79	1.21E-02	2.20	1.19	4.07
octadecanedioate (C18)	Fatty Acid, Dicarboxylate	Lipid	0.64	1.36E-02	1.90	1.14	3.15
N4-acetylcytidine	Pyrimidine Metabolism, Cytidine containing	Nucleotide	0.65	1.37E-02	1.92	1.14	3.23
tetradecadienoate (14:2)*	Long Chain Polyunsaturated Fatty Acid (n3 and n6)	Lipid	0.59	1.55E-02	1.81	1.12	2.93
alliin	Food Component/Plant	Xenobiotics	-0.38	1.57E-02	0.68	0.50	0.93
hydroxyasparagine	Alanine and Aspartate Metabolism	Amino Acid	0.95	1.75E-02	2.59	1.18	5.68
hydroxyproline	Urea cycle; Arginine and Proline Metabolism	Amino Acid	0.84	1.95E-02	2.32	1.15	4.70
dodecadienoate (12:2)*	Fatty Acid, Dicarboxylate	Lipid	0.58	1.98E-02	1.79	1.10	2.93
sphingomyelin (d18:1/22:2, d18:2/22:1, d16:1/24:2)*	Sphingomyelins	Lipid	0.93	2.03E-02	2.54	1.16	5.60
4-vinylphenol sulfate	Benzoate Metabolism	Xenobiotics	-0.40	2.60E-02	0.67	0.47	0.95
sulfate*	Chemical	Xenobiotics	0.93	2.61E-02	2.53	1.12	5.72
2-hydroxystearate	Fatty Acid, Monohydroxy	Lipid	0.70	2.81E-02	2.02	1.08	3.77
5-methylthioadenosine (MTA)	Polyamine Metabolism	Amino Acid	0.51	2.84E-02	1.66	1.06	2.62
pregnenetriol disulfate*	Pregnenolone Steroids	Lipid	0.58	2.92E-02	1.79	1.06	3.01
1-arachidonylglycerol (20:4)	Monoacylglycerol	Lipid	0.43	2.99E-02	1.54	1.04	2.27
docosatrienoate (22:3n3)	Long Chain Polyunsaturated Fatty Acid (n3 and n6)	Lipid	0.47	3.20E-02	1.59	1.04	2.44
heptanoate (7:0)	Medium Chain Fatty Acid	Lipid	0.77	3.26E-02	2.16	1.07	4.38
retinol (Vitamin A)	Vitamin A Metabolism	Cofactors and Vitamins	-0.74	3.27E-02	0.48	0.24	0.94
N-acetylalanine	Alanine and Aspartate Metabolism	Amino Acid	0.99	3.30E-02	2.69	1.08	6.68
urate	Purine Metabolism, (Hypo)Xanthine/Inosine containing	Nucleotide	0.85	3.35E-02	2.34	1.07	5.12
1-oleoyl-2-docosahexaenoyl-GPC (18:1/22:6)*	Phosphatidylcholine (PC)	Lipid	-0.81	3.78E-02	0.45	0.21	0.96
oleate/vaccenate (18:1)	Long Chain Monounsaturated Fatty Acid	Lipid	0.49	3.95E-02	1.63	1.02	2.58

Metabolite	Sub-pathway	Super-pathway	Beta estimate	p-value	Odds ratio	95% CI lower bound	95% CI upper bound
2-hydroxybutyrate/2-hydroxyisobutyrate	Glutathione Metabolism	Amino Acid	0.62	4.00E-02	1.86	1.03	3.36
hydroxypalmitoyl sphingomyelin (d18:1/16:0(OH))	Sphingomyelins	Lipid	0.79	4.11E-02	2.19	1.03	4.66
7-HOCA	Sterol	Lipid	0.71	4.17E-02	2.04	1.03	4.03
cis-4-decenoylcarnitine (C10:1)	Fatty Acid Metabolism (Acyl Carnitine, Monounsaturated)	Lipid	0.50	4.25E-02	1.65	1.02	2.67
linoleate (18:2n6)	Long Chain Polyunsaturated Fatty Acid (n3 and n6)	Lipid	0.48	4.56E-02	1.61	1.01	2.58
dihomolinoleate (20:2n6)	Long Chain Polyunsaturated Fatty Acid (n3 and n6)	Lipid	0.52	5.00E-02	1.69	1.00	2.85

*: indicates a compound that has not been confirmed based on a standard, but we are confident in its identity.

Abbreviations: VDAART, Vitamin D Antenatal Asthma Reduction Trial.

Supplementary Table S7. Maternal baseline (10–18 weeks gestation) metabolites with *p*-values < 0.05 for their interaction with child sex in association with child asthma/recurrent wheeze status by age 3 in VDAART (sorted by interaction term *p*-values)

Metabolite	Sub-pathway	Super-pathway	Beta estimate	Interaction term p-value
thioproline	Chemical	Xenobiotics	1.72	2.58E-03
kynurenine	Tryptophan Metabolism	Amino Acid	1.63	4.22E-03
3-methylxanthine	Xanthine Metabolism	Xenobiotics	0.68	6.19E-03
theobromine	Xanthine Metabolism	Xenobiotics	0.47	7.72E-03
glyco-beta-muricholate	Primary Bile Acid Metabolism	Lipid	-0.67	1.01E-02
pseudouridine	Pyrimidine Metabolism, Uracil containing	Nucleotide	1.72	1.04E-02
1-palmitoyl-2-palmitoleoyl-GPC (16:0/16:1)*	Phosphatidylcholine (PC)	Lipid	1.04	1.08E-02
N-oleoylserine	Endocannabinoid	Lipid	-1.02	1.43E-02
gulonate*	Ascorbate and Aldarate Metabolism	Cofactors and Vitamins	0.58	1.56E-02
cysteinylglycine disulfide*	Glutathione Metabolism	Amino Acid	1.23	1.56E-02
1-stearoyl-2-oleoyl-GPS (18:0/18:1)	Phosphatidylserine (PS)	Lipid	0.79	1.72E-02
cysteine	Methionine, Cysteine, SAM and Taurine Metabolism	Amino Acid	1.01	1.98E-02
1-(1-enyl-palmitoyl)-2-linoleoyl-GPC (P-16:0/18:2)*	Plasmalogen	Lipid	-1.28	2.25E-02
5alpha-pregnan-3beta,20beta-diol monosulfate (1)	Progestin Steroids	Lipid	-1.96	2.29E-02
isoursodeoxycholate	Secondary Bile Acid Metabolism	Lipid	-0.56	2.42E-02
3-amino-2-piperidone	Urea cycle; Arginine and Proline Metabolism	Amino Acid	-0.85	2.58E-02
hexadecenedioate (C16:1-DC)*	Fatty Acid, Dicarboxylate	Lipid	-0.78	2.66E-02
hydroxy-N6,N6,N6-trimethyllysine*	Lysine Metabolism	Amino Acid	0.75	3.05E-02
1-(1-enyl-palmitoyl)-GPC (P-16:0)*	Lysoplasmalogen	Lipid	-0.93	3.06E-02
taurolithocholate 3-sulfate	Secondary Bile Acid Metabolism	Lipid	-0.61	3.65E-02
cysteinylglycine	Glutathione Metabolism	Amino Acid	0.60	3.82E-02
C-glycosyltryptophan	Tryptophan Metabolism	Amino Acid	1.18	4.01E-02
3beta-hydroxy-5-cholestenoate	Sterol	Lipid	-0.94	4.10E-02
12,13-DiHOME	Fatty Acid, Dihydroxy	Lipid	-0.57	4.22E-02

Metabolite	Sub-pathway	Super-pathway	Beta estimate	Interaction term p-value
maltotriose	Glycogen Metabolism	Carbohydrate	0.38	4.47E-02
maltose	Glycogen Metabolism	Carbohydrate	0.45	4.75E-02

*: indicates a compound that has not been confirmed based on a standard, but we are confident in its identity.

Supplementary Table S8. Maternal third trimester (32–38 weeks gestation) metabolites with *p*-values < 0.05 for their interaction with child sex in association with child asthma/recurrent wheeze status by age 3 in VDAART (sorted by interaction term *p*-values)

Metabolite	Sub-pathway	Super-pathway	Beta estimate	Interaction term p-value
quinolinate	Nicotinate and Nicotinamide Metabolism	Cofactors and Vitamins	1.58	2.53E-04
2-hydroxyhippurate (salicylurate)	Benzoate Metabolism	Xenobiotics	0.83	9.79E-04
salicylate	Drug - Topical Agents	Xenobiotics	1.03	1.85E-03
3-(3-amino-3-carboxypropyl)uridine*	Pyrimidine Metabolism, Uracil containing	Nucleotide	1.46	2.77E-03
C-glycosyltryptophan	Tryptophan Metabolism	Amino Acid	1.68	5.20E-03
5,6-dihydrouridine	Pyrimidine Metabolism, Uracil containing	Nucleotide	1.77	5.78E-03
kynurenine	Tryptophan Metabolism	Amino Acid	1.50	6.25E-03
N2,N2-dimethylguanosine	Purine Metabolism, Guanine containing	Nucleotide	1.60	7.16E-03
1-stearoyl-2-docosahexaenoyl-GPE (18:0/22:6)*	Phosphatidylethanolamine (PE)	Lipid	1.32	7.47E-03
N6-carbamoylthreonyladenosine	Purine Metabolism, Adenine containing	Nucleotide	1.70	7.68E-03
hexadecenedioate (C16:1-DC)*	Fatty Acid, Dicarboxylate	Lipid	-0.98	9.96E-03
aconitate [cis or trans]	TCA Cycle	Energy	1.58	1.13E-02
pseudouridine	Pyrimidine Metabolism, Uracil containing	Nucleotide	1.67	1.30E-02
ADP	Purine Metabolism, Adenine containing	Nucleotide	0.71	1.35E-02
3-methylxanthine	Xanthine Metabolism	Xenobiotics	0.53	1.41E-02
sphingadienine	Sphingolipid Synthesis	Lipid	0.94	1.56E-02
7-methylguanine	Purine Metabolism, Guanine containing	Nucleotide	1.57	1.56E-02
1,7-dimethylurate	Xanthine Metabolism	Xenobiotics	0.53	1.67E-02
hydroxyasparagine	Alanine and Aspartate Metabolism	Amino Acid	1.44	1.73E-02
urate	Purine Metabolism, (Hypo)Xanthine/Inosine containing	Nucleotide	1.42	1.77E-02
phosphoethanolamine (PE)	Phospholipid Metabolism	Lipid	0.81	2.06E-02
beta-cryptoxanthin	Vitamin A Metabolism	Cofactors and Vitamins	-0.82	2.08E-02
2-hydroxylaurate	Fatty Acid, Monohydroxy	Lipid	-1.15	2.22E-02
caffeine	Xanthine Metabolism	Xenobiotics	0.46	2.27E-02

Metabolite	Sub-pathway	Super-pathway	Beta estimate	Interaction term p-value
N-acetylneuraminate	Aminosugar Metabolism	Carbohydrate	1.10	2.27E-02
orotidine	Pyrimidine Metabolism, Orotate containing	Nucleotide	1.15	2.36E-02
eicosenedioate (C20:1-DC)*	Fatty Acid, Dicarboxylate	Lipid	-0.84	2.40E-02
kynurenate	Tryptophan Metabolism	Amino Acid	1.01	2.62E-02
cortolone glucuronide (1)	Corticosteroids	Lipid	0.75	2.71E-02
sphingosine	Sphingosines	Lipid	0.97	2.72E-02
N4-acetylcytidine	Pyrimidine Metabolism, Cytidine containing	Nucleotide	0.80	2.95E-02
dodecanedioate (C12)	Fatty Acid, Dicarboxylate	Lipid	-0.68	3.28E-02
theophylline	Xanthine Metabolism	Xenobiotics	0.48	3.33E-02
1-palmitoyl-2-docosahexaenoyl-GPE (16:0/22:6)*	Phosphatidylethanolamine (PE)	Lipid	1.03	3.51E-02
palmitoleoylcarnitine (C16:1)*	Fatty Acid Metabolism (Acyl Carnitine, Monounsaturated)	Lipid	0.77	3.55E-02
N-acetyltryptophan	Tryptophan Metabolism	Amino Acid	0.59	3.70E-02
creatinine	Creatine Metabolism	Amino Acid	1.58	3.73E-02
theobromine	Xanthine Metabolism	Xenobiotics	0.47	3.76E-02
dihomo-linolenoylcarnitine (C20:3n3 or 6)*	Fatty Acid Metabolism (Acyl Carnitine, Polyunsaturated)	Lipid	0.68	3.96E-02
vanillylmandelate (VMA)	Tyrosine Metabolism	Amino Acid	0.82	3.96E-02
octadecadienedioate (C18:2-DC)*	Fatty Acid, Dicarboxylate	Lipid	-0.69	4.08E-02
1-arachidonylglycerol (20:4)	Monoacylglycerol	Lipid	0.56	4.15E-02
pyridoxate	Vitamin B6 Metabolism	Cofactors and Vitamins	-0.61	4.18E-02
docosatrienoate (22:3n3)	Long Chain Polyunsaturated Fatty Acid (n3 and n6)	Lipid	0.62	4.34E-02
gamma-glutamylhistidine	Gamma-glutamyl Amino Acid	Peptide	0.89	4.36E-02
carotene diol (3)	Vitamin A Metabolism	Cofactors and Vitamins	-0.85	4.47E-02

*: indicates a compound that has not been confirmed based on a standard, but we are confident in its identity.

Metabolomic profiling in the Vitamin D Antenatal Asthma Reduction Trial (VDAART)

Metabolon ultrahigh-performance liquid chromatography (UPLC) – tandem mass spectrometry (MS/MS) platform method [1]

Sample Preparation: Samples were prepared using the automated MicroLab STAR® system from Hamilton Company. Several recovery standards were added prior to the first step in the extraction process for QC purposes. To remove protein, dissociate small molecules bound to protein or trapped in the precipitated protein matrix, and to recover chemically diverse metabolites, proteins were precipitated with methanol under vigorous shaking for 2 min (Glen Mills GenoGrinder 2000) followed by centrifugation. The resulting extract was divided into five fractions: two for analysis by two separate reverse phase (RP)/UPLC-MS/MS methods with positive ion mode electrospray ionization (ESI), one for analysis by RP/UPLC-MS/MS with negative ion mode ESI, one for analysis by HILIC/UPLC-MS/MS with negative ion mode ESI, and one sample was reserved for backup. Samples were placed briefly on a TurboVap® (Zymark) to remove the organic solvent. The sample extracts were stored overnight under nitrogen before preparation for analysis.

QA/QC: Several types of controls were analyzed in concert with the experimental samples: a well-characterized, pooled matrix sample generated from a representative, large number of individuals served as a technical replicate throughout the data set; extracted water samples served as process blanks; and a cocktail of QC standards that were carefully chosen not to interfere with the measurement of endogenous compounds were spiked into every analyzed sample to allow instrument performance monitoring and aid in chromatographic alignment. Instrument variability was determined by calculating the median relative standard deviation (RSD) for the standards that were added to each sample prior to injection into the mass spectrometers. Overall process variability was determined by calculating the median RSD for all endogenous metabolites (i.e., non-instrument standards) present in 100% of the pooled matrix samples. Experimental samples were randomized across the platform run with QC samples spaced evenly among the injections.

Ultrahigh Performance Liquid Chromatography – Tandem Mass Spectrometry (UPLC-MS/MS): All methods utilized a Waters ACQUITY ultra-performance liquid chromatography (UPLC) and a Thermo Scientific Q-Exactive high resolution/accurate mass spectrometer interfaced with a heated electrospray ionization (HESI-II) source and Orbitrap mass analyzer operated at 35,000 mass resolution. The sample extract was dried then reconstituted in solvents compatible to each of the four methods. Each reconstitution solvent contained a series of standards at fixed concentrations to ensure injection and chromatographic consistency. One aliquot was analyzed using acidic positive ion conditions, chromatographically optimized for more hydrophilic compounds. In this method, the extract was gradient eluted from a C18 column (Waters UPLC BEH C18-2.1x100 mm, 1.7 µm) using water and methanol, containing 0.05% perfluoropentanoic acid (PFPA) and 0.1% formic acid (FA). Another aliquot was also analyzed using acidic positive ion conditions, however it was chromatographically optimized for more hydrophobic compounds. In this method, the extract was gradient eluted from the same afore mentioned C18 column using methanol, acetonitrile, water, 0.05% PFPA and 0.01% FA and was operated at an overall higher organic content. Another aliquot was analyzed using basic negative ion optimized conditions using a separate dedicated C18 column. The basic extracts were gradient eluted from the column using methanol and water, however with 6.5 mM Ammonium Bicarbonate at pH 8. The fourth aliquot was analyzed via negative ionization following elution from a HILIC column (Waters UPLC BEH Amide 2.1x150 mm, 1.7 µm) using a gradient consisting of water and acetonitrile with 10mM Ammonium Formate, pH 10.8. The MS analysis alternated between MS and data-dependent MSn scans using dynamic exclusion. The scan range varied slighted between methods but covered 70-1000 m/z. Raw data files were archived and extracted as described below.

Data Extraction and Compound Identification: Raw data was extracted, peak-identified and QC processed using Metabolon's hardware and software. These systems are built on a web-service platform utilizing Microsoft's .NET technologies, which run on high-performance application servers and fiber-channel storage arrays in clusters to provide active failover and load-balancing. Compounds were identified by comparison to library entries of purified standards or recurrent unknown entities. Metabolon maintains a library based on authenticated standards that contains the retention time/index (RI), mass to charge ratio (m/z), and chromatographic data (including MS/MS spectral data) on all molecules present in the library. Furthermore, biochemical identifications are based on three criteria: retention index within a narrow RI window of the proposed identification, accurate mass match to the library +/- 10 ppm, and the MS/MS forward and reverse scores between the experimental data and authentic standards. The MS/MS scores are based on a comparison of the ions present in the experimental spectrum to the ions present in the library spectrum. While there may be similarities between these molecules based on one of these factors, the use of all three data points can be utilized to distinguish and differentiate biochemicals. More than 4,500 commercially available purified standard compounds have been acquired and registered into LIMS for analysis on all platforms for determination of their analytical characteristics. Additional mass spectral entries have been created for structurally unnamed biochemicals, which have been identified by virtue of their recurrent nature (both chromatographic and mass spectral). These compounds have the potential to be identified by future acquisition of a matching purified standard or by classical structural analysis.

Curation: A variety of curation procedures were carried out to ensure that a high quality data set was made available for statistical analysis and data interpretation. The QC and curation processes were designed to ensure accurate and consistent identification of true chemical entities, and to remove those representing system artifacts, mis-assignments, and background noise. Metabolon data analysts use proprietary visualization and interpretation software to confirm the consistency of peak identification among the various samples. Library matches for each compound were checked for each sample and corrected if necessary.

Metabolite Quantification and Data Normalization: Peaks were quantified using area-under-the-curve. A data normalization step was performed to correct variation resulting from instrument inter-day tuning differences by registering the medians to equal one and normalizing each data point proportionately. For this study, two distinct sample sets were merged that contained disparity in the proportions of control group composition, precluding run day normalization. Accordingly, datasets were merged by setting the medians of the control groups in the two datasets to be equal. Because sampling was kept consistent across both sample sets, this mitigates concerns that this approach artificially decreases the variance of this control group by forcing the medians to be equal.

Data processing pipeline

We calculated missingness across each metabolite and each sample. Metabolites missing ≥ 75% were excluded. Missing values were imputed as half the minimum value across all samples for each metabolite. We examined the resulting plots of principal component analysis (PCA). Interquartile range (IQR) and skewness of each metabolite were also computed. All metabolites were subsequently log-10 transformed and *pareto*-scaled, and IQR and skewness were re-calculated after transformation. PCA was performed again, and distribution of PCs according to demographic variables were examined.

References

1. Evans, A.M.; DeHaven, C.D.; Barrett, T.; Mitchell, M.; Milgram, E. Integrated, nontargeted ultrahigh performance liquid chromatography/electrospray ionization tandem mass spectrometry platform for the identification and relative quantification of the small-molecule complement of biological systems. *Anal Chem* **2009**, *81*, 6656-6667, doi:10.1021/ac901536h.
